# Efficiently prepared ephedrine alkaloids-free Ephedra Herb extract: a putative marker and antiproliferative effects

**DOI:** 10.1007/s11418-016-0977-1

**Published:** 2016-03-14

**Authors:** Naohiro Oshima, Tadatoshi Yamashita, Sumiko Hyuga, Masashi Hyuga, Hiroyuki Kamakura, Morio Yoshimura, Takuro Maruyama, Takashi Hakamatsuka, Yoshiaki Amakura, Toshihiko Hanawa, Yukihiro Goda

**Affiliations:** National Institute of Health Sciences, 1-18-1 Kamiyoga, Setagaya-ku, Tokyo, 158-8501 Japan; Department of Pharmaceutical Sciences, International University of Health and Welfare, 2600-1, Kitakanemaru, Ohtawara city, Tochigi 324-8501 Japan; TOKIWA Phytochemical Co., Ltd., 158 Kinoko, Sakura-shi, Chiba, 285-0801 Japan; Department of Clinical Research, Oriental Medicine Research Center of Kitasato University, 5-9-1 Shirokane, Minato-ku, Tokyo, 108-8642 Japan; Department of Pharmacognosy, College of Pharmaceutical Sciences, Matsuyama University, 4-2 Bunkyo-cho, Matsuyama, Ehime 790-8578 Japan

**Keywords:** Ephedra Herb, Ephedrine alkaloids-free Ephedra Herb extract, Chemical composition, Herbacetin, Antiproliferative effect

## Abstract

Ephedrine alkaloids (EAs) have been considered the main pharmacologically active substances in Ephedra Herb (麻黄, Mao; EH) since they were first identified by Prof. N. Nagai, and are known to induce palpitation, hypertension, insomnia, and dysuria as side effects. Therefore, the administration of drugs containing EH to patients with cardiovascular-related diseases is severely contraindicated. While our previous studies suggest that some of the effects of EH may not be due to EAs, considering their side effects would be expedient to develop a new EAs-free EH extract (EFE). Here, we established a preparation method for EFE and revealed its chemical composition, including the content of herbacetin, a flavonoid aglycon present in EH and a potential putative marker for EFE quality control. In addition, we showed the antiproliferative effects of EFE against the H1975 non-small cell lung cancer (NSCLC) cell line. EFE was prepared from EH extract using the ion exchange resin SK-1B. LC/Orbitrap MS analysis revealed the removal of EAs, 6-methoxykynurenic acid, and 6-hydroxykynurenic acid from the original extract. Quantitative analysis of herbacetin using LC/MS in acid-hydrolyzed EFE showed that its content was 0.104 %. Although several alkaloidal constituents were removed from EH extract, the antiproliferative effect of EFE against H1975 cells was comparable to that of EH extract. These results indicate that EFE retained the anticancer effect of EH and demonstrated its potential for future development as a new herbal medicine with reduced side effects.

**Electronic supplementary material:**

The online version of this article (doi:10.1007/s11418-016-0977-1) contains supplementary material, which is available to authorized users.

## Introduction

Ephedra Herb (EH) is officially defined as the terrestrial stem of *Ephedra sinica* Stapf, *Ephedra intermedia* Schrenk et C. A. Meyer, or *Ephedra equisetina* Bunge (Ephedraceae) in the Japanese Pharmacopoeia 16th edition (JP16) [1]. EH is a component of Kampo (Japanese traditional herbal medicine) formulae for the treatment of headaches, bronchial asthma, nasal inflammation, and the common cold, and is reported to have anti-inflammatory [2], antitussive [3], and anti-influenza activities [4].

Ephedrine alkaloids (EAs) were isolated as principal ingredients in EH by Prof. Nagayoshi Nagai in 1885 [5]. Miura [6] showed that ephedrine has mydriatic action in the rabbits. Then, Amatsu and Kubota [7] reported that ephedrine raised the blood pressure by contraction of the peripheral vessels following intravenous (i.v.) injection in dogs. Chen and Schmidt [8] found that ephedrine showed circulatory stimulatory effects when it was orally administered. Furthermore, MacDermot [9] revealed that the injection of ephedrine into patients with bronchial asthma showed beneficial effects. EAs have considerable pharmacological activities, and are believed to be the principal active ingredients in EH. The content of EAs in EH are regulated in the JP16. However, EAs are known to induce palpitation, hypertension, insomnia, and dysuria as major side effects. Therefore, the administration of EAs-containing drugs to patients with cardiovascular-related diseases is severely contraindicated.

Previously, we found that EH extract impaired hepatocyte growth factor (HGF)-induced cancer cell motility, likely by suppressing the HGF-c-Met signaling pathway [10], since dysregulation of this pathway promotes tumor formation, growth, progression, metastasis, and therapeutic resistance [11, 12]. Therefore, EH may have applications in cancer therapy as a novel c-Met inhibitor. Recently, we revealed that herbacetin, a flavonoid aglycon in EH, inhibited HGF/c-Met/Akt signal and HGF-induced motility of human MDA-MB-231 breast cancer cells [13]. In addition, we found that herbacetin had analgesic effects in the formalin test [14]. These results indicate that some of the pharmacological effects of EH may not be due to EAs and, therefore, the prospect of preparing an EAs-free EH extract (EFE) as a new and potentially safer natural medicine without the side effects associated with EAs appealed to us.

Therefore, to achieve the aim of this present study, which was the production of a clinically useful EH extract with none of the side effects associated with EAs, we developed an efficient method for preparing EFE from EH extract. Furthermore, we clarified the chemical composition of the EFE and analyzed the herbacetin content as a candidate marker using LC–MS because EFE contains no EAs, which are markers for the quantitative assay of EH stipulated by the JP16. In addition, we examined its antiproliferative effects against the H1975 non-small cell lung cancer (NSCLC) cell line.

## Materials and methods

### Materials and reagents

EH (JP16 grade) originally produced from *E. sinica* was purchased from Uchida Wakanyaku Co., Ltd. The authentic EAs used were: ephedrine, purchased from Dainippon Pharma Co., Ltd.; methylephedrine and pseudoephedrine, from Alps Pharmaceutical Ind., Co., Ltd.; and norephedrine from Tokyo Chemical Industry Co. Ltd. 6-Methoxykynurenic acid was purchased from Chemicia Scientific, LLC. *trans*-Cinnamic acid was purchased from Wako Co., while herbacetin 7-*O*-neohesperidoside and herbacetin 7-*O*-glucoside were isolated from EH [15]. Herbacetin and apigenin were purchased from ChromaDex Co. and Wako Co., respectively.

### General procedures

Unless otherwise noted, the following instruments and conditions were used. The ^1^H-NMR, ^13^C-NMR, and 2D-NMR spectra were recorded using an ECA-800 or ECA-600 spectrometer (JEOL), and chemical shifts were expressed in *δ* (ppm) with tetramethylsilane (TMS) as the reference standard. The LC/Orbitrap MS analysis was performed using an LC-20A UFLC system (Shimadzu) equipped with the LTQ Orbitrap XL mass spectrometer (Thermo Fisher Scientific). The UPLC/MS analysis was performed using a Xevo TQD UPLC/MS system (Waters). The LC/MS analysis was performed using an LC-20A UFLC system equipped with an LCMS-2020 mass spectrometer (Shimadzu).

### Selection of optimal cation exchange resin for EFE preparation

#### Sample preparation

EH (10 g) was extracted with hot water (100 ml) for 1 h at 95 °C. After filtration, the extract volume was adjusted to 100 ml with water and 2 ml was exposed to each cation exchange resin (2 ml) shown in Table 1, stirred for 10 s, and then the solution was left to stand for 1 h. The supernatant (0.5 ml) volume was adjusted to 5 ml with MeOH and subsequently used as the sample solution for the HPLC analysis.

**Table 1:** Cation exchange resins

	Resins	Class	Manufacturers
A	WK10	Weak	Mitsubishi Chemical Co., Japan
B	WK11	Weak	Mitsubishi Chemical Co., Japan
C	WK20	Weak	Mitsubishi Chemical Co., Japan
D	WK40L	Weak	Mitsubishi Chemical Co., Japan
E	SK104	Strong	Mitsubishi Chemical Co., Japan
F	SK110	Strong	Mitsubishi Chemical Co., Japan
G	SK1B	Strong	Mitsubishi Chemical Co., Japan
H	UBK530	Strong	Mitsubishi Chemical Co., Japan
I	UBK12	Strong	Mitsubishi Chemical Co., Japan
J	PK216	Strong	Mitsubishi Chemical Co., Japan
K	IR120B	Strong	Organo Co., Japan
L	FPC3500	Strong	Organo Co., Japan
M	1060H	Strong	Organo Co., Japan

#### Quantitative analyses of ephedrine alkaloids

The HPLC analysis was performed using the HITACHI HPLC system (pump, L-2130; degasser, L-2130; auto-sampler, L-2200; column oven, L-2300; and diode array detector, L-2450). The analytical conditions were as follows [1]: column, SHISEIDO AG 120 (4.6 mm i.d. × 150 mm; 5 μm, Shiseido, Tokyo); mobile phase, sodium lauryl sulfate (5 g) dissolved in acetonitrile (MeCN, 350 ml), followed by the addition of water (650 ml) and phosphoric acid (1 ml); flow rate, 0.8 ml/min; column oven temperature, 45 °C; injection volume, 10 μl; and monitoring wavelength, 210 nm.

#### Preparation of EFE

EH (10 kg) was added to water (100 l), extracted at 95 °C for 1 h, and the extract was filtered through the SK-1B ion exchange resin (10 l, Mitsubishi Chemical Co.), which was treated with 1 M HCl (30 l) and water (100 l) prior to use, at a flow rate of 0.5 l/min, and then the resin was washed with water (10 l). The unadsorbed fraction (110 l) was adjusted to pH 5 with 5 % aqueous sodium bicarbonate (NaHCO_3_aq., 6 l). The solution was evaporated under reduced pressure to obtain EFE (1.2 kg, yield 12.0 %).

### Analysis of the chemical composition of EFE

#### Preparation of sample solution

EH (200 g) was added to water (2000 ml), extracted at 95 °C for 1 h, filtered, and then the residue was washed with water (200 ml). The extract was centrifuged at 1800 × g for 10 min, and then half of the supernatant was concentrated under reduced pressure to obtain the EH extract (14.1 g), while the other half was passed through the SK-1B ion exchange resin (100 ml), which was treated with 1 M HCl (30 ml) and water (100 ml) prior to use, and then the resin was washed with water (100 ml). The unadsorbed fraction (1100 ml) was adjusted to pH 5 using 5 % NaHCO_3_aq. (60 ml), and then the solution was evaporated under reduced pressure to obtain EFE (11.8 g). Each extract batch was adjusted to a concentration of 5 mg/ml with 50 % aqueous MeOH (MeOHaq.), filtered using a 0.45-μm filter, and then subsequently used as the sample solutions.

#### Analysis of LC/Orbitrap MS

The LC/Orbitrap MS analytical conditions were as follows: column, Inertsil ODS-3 (2.1 mm i.d. × 150 mm, 5 μm; GL Sciences); mobile phase, 0.1 % formic acid (HCOOH) in water (A)–0.1 % HCOOH in MeOH (B) in a gradient mode of 5 % B (0–10 min) → 75 % B (70 min) → 100 % B (80 min) → 100 % B (90 min) → 5 % B (90.01 min) → 5 % B (95 min); injection volume, 1 μl; flow rate, 0.2 ml/min; column oven temperature, 40 °C; and photodiode array (PDA) (200–400 nm). Furthermore, the MS conditions were: interface, electrospray ionization (ESI) positive/negative; source voltage, 4.0 kV; capillary voltage, 10 V; source temperature, 300 °C; sheath and auxiliary gas flow rates, 50 and 25, respectively; scan range, *m*/*z* 50–2000; and mass resolution, 30,000 full width.

### Analysis of UPLC/MS

The UPLC analytical conditions were: column, Inertsil ODS-3 (2.1 mm i.d. × 150 mm, 5 μm; GL Sciences); mobile phase, 0.1 % HCOOH in water (A)–0.1 % HCOOH in MeOH (B) in a gradient mode of 5 % B (0 min) → 50 % B (40 min) → 100 % B (50 min) → 100 % B (55 min) → 5 % B (55.1 min) → 5 % B (60 min); injection volume, 1 μl; flow rate, 0.2 ml/min; and PDA (200–400 nm). In addition, the MS conditions were: interface, ESI positive/negative; capillary voltage, 4.5 kV; source and desolvation temperatures, 150 and 400 °C, respectively; desolvation gas flow, 800 l/h; cone voltage, 50 V; cone gas flow, 50 l/h; and scan range, *m*/*z* 100–1200.

### Synthesis of 6-hydroxykynurenic acid (2)

6-Methoxykynurenic acid (48.4 mg) was dissolved in ethylene glycol (5 ml) and potassium hydroxide (KOH, 1 g) was added, followed by refluxing for 4.5 h. The reaction mixture was carefully neutralized with HCl under cooling conditions, separated using LH-20, and preparative TLC was performed to obtain **2** (10.7 mg).

*6-Hydroxykynurenic acid (****2****)*: White crystal, ^1^H-NMR (800 MHz, DMSO-*d*_6_): *δ*_H_ 6.35 (1H, s, H-3), 7.31 (1H, d, *J* = 3.2 Hz, H-5), 7.05 (1H, dd, *J* = 8.8, 3.2 Hz, H-7), 7.77 (1H, d, *J* = 8.8 Hz, H-8), 9.73 (1H, br s), 11.2 (1H, s). ^13^C-NMR (200 MHz, DMSO-*d*_6_): *δ*_C_ 146.9 (C-2), 106.5 (C-3), 163.7 (C-4), 127.4 (C-4a), 107.5 (C-5), 153.8 (C-6), 122.1 (C-7), 121.3 (C-8), 123.5 (C-8a), 166.8 (COOH). ESI-MS: *m*/*z* 206 [M+H]^+^.

### Quantitative analysis of herbacetin 7-*O*-neohesperidoside

#### Calibration curve

The standard stock solution was prepared by accurately weighing an adequate amount of herbacetin 7-*O*-neohesperidoside and dissolving it in 50 % MeOH. The working standard solutions were prepared by diluting the stock solution with 50 % MeOH to give six graded concentrations of 0.1, 0.5, 1.0, 5.0, and 50.0 µg/ml. Each standard solution was analyzed in sextuplicate, and the regression equation was calculated in the form *y* = A*x* + B.

#### Sample preparation

The EFE sample for the LC/MS analysis was prepared by adjusting the concentration to 1 mg/ml with 50 % MeOH and filtering with a 0.45-μm filter.

#### Quantitative analysis

The chromatographic analytical conditions were: column, Inertsil ODS-3 (150 mm × 2.1 mm i.d., 5 μm; GL Sciences); mobile phase, 0.1 % HCOOH in water (A)–MeCN (B) in a gradient mode of 0 % B (0 min) → 40 % B (50 min) → 100 % B (60 min) → 100 % B (70 min) → 0 % B (70.01 min) → 0 % B (75 min); injection volume, 1 μl; and flow rate, 0.2 ml/min. The MS conditions were: interface, ESI negative; nebulizer gas flow, 1.5 l/min; drying gas flow, 10 l/min; curved desolvation line (CDL) and heat block temperature, 250 and 200 °C, respectively; detector and interface voltage, 120 and 4.5/−4.5 kV, respectively; interface current, 0.6 μA; and MS range, *m*/*z* 609 (selective ion monitoring, SIM). The EFE sample was analyzed in sextuplicate.

### Quantitative analysis of herbacetin

#### Acid hydrolysis of EFE

HCl (6 M, 5.0 ml) was correctly added to EFE (5.0 mg) and the reaction mixture was heated at 70 °C for 6 h, followed by separation on a CHP 20P column (*ϕ* 2 × 20 cm). After washing with water, the MeOH eluted fraction was evaporated under reduced pressure, the residue was dissolved in MeOH, and the volume was adjusted to 5.0 ml. Then, 4.5 ml was added to 0.5 ml of apigenin solution (1 mg/ml, MeOH), followed by filtration with a 0.45-μm filter.

#### Calibration curve

The calibration curve for herbacetin was constructed similarly to that for herbacetin 7-*O*-neohesperidoside, with graded concentrations of 0.018, 0.045, 0.09, 0.18, 0.45, 0.9, and 1.8 μg/ml. Furthermore, apigenin was used as the internal standard at a final concentration of 100 µg/ml.

#### Quantitative analysis

The chromatographic analytical conditions were: column, Xbridge C18 (2.1 mm i.d. × 100 mm, 3.5 μm; Waters); mobile phase, 0.1 % HCOOH in water (A)–MeCN (B) in a gradient mode of 0 % B (0 min) → 40% B (50 min) → 100 % B (60 min) → 100 % B (70 min) → 0 % B (70.01 min) → 0 % B (75 min); injection volume, 1 μl; and flow rate, 0.2 ml/min. The MS analytical conditions were: interface, ESI negative; nebulizer and drying gas flow, 1.5 and 10 l/min, respectively; CDL and heat block temperature, 250 and 200 °C, respectively; detector and interface voltage, 120 and 4.5/−4.5 kV, respectively; interface current, 0.6 μA; and MS range, *m*/*z* 301 (SIM). The EFE sample was analyzed in sextuplicate.

### Antiproliferative effect

The H1975 NSCLC cell line was obtained from the American Type Culture Collection (ATCC, Manassas, VA, USA). The cells were suspended at a density of 2 × 10^3^ cells in 100 μl Roswell Park Memorial Institute (RPMI) 1640 medium (Invitrogen Co.) containing 10 % fetal calf serum (FCS, Sigma-Aldrich), with or without 50–200 μg/ml of EH extract or EFE in each well of a 96-well plate and incubated at 37 °C for 72 h. To each well was added 10 μl of Cell Counting Kit-8 solution (Dojindo Co.), and after a 2-h incubation at 37 °C, the absorbance of the formazan generated in each well was measured at 450 nm using an iMark plate reader (Bio-Rad Laboratories, Inc.). The IC_50_ value was calculated using a four-parameter logistic model (Prism 5.0, GraphPad software).

## Results and discussion

**Fig. 1:**
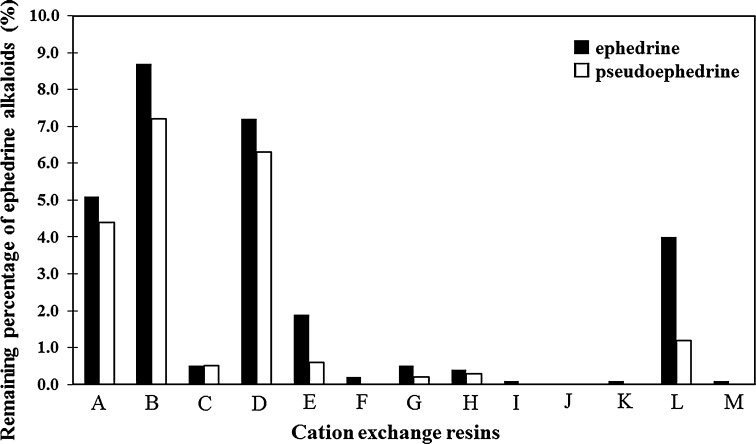
Residual ratio of ephedrine alkaloids (EAs) exposed to each cation exchange resin (%). Amounts of ephedrine and pseudoephedrine in Ephedra Herb (EH) corresponding to 100 % of the vertical axis were 74.0 and 22.1 mg, respectively

We used cation exchange resins to remove EAs from the EH extract and efficiently prepared the EFE. The prototype EFEs were formulated using the 13 cation exchange resins shown in Table 1, and the residual EAs in each prototype were quantitatively determined. The results revealed that the residual ratios of EAs in the resins C-, F–K-, and M-treated prototypes were less than 1 % (Fig. 1) and, therefore, we focused on resin G (SK1B) considering cost efficiency, in preparing the EFE for practical clinical use. By using resin G, the yield of the EFE prepared for subsequent experiments from the EH extract was high (1.2 kg; y. 12.0 % from EH).

**Fig. 2:**
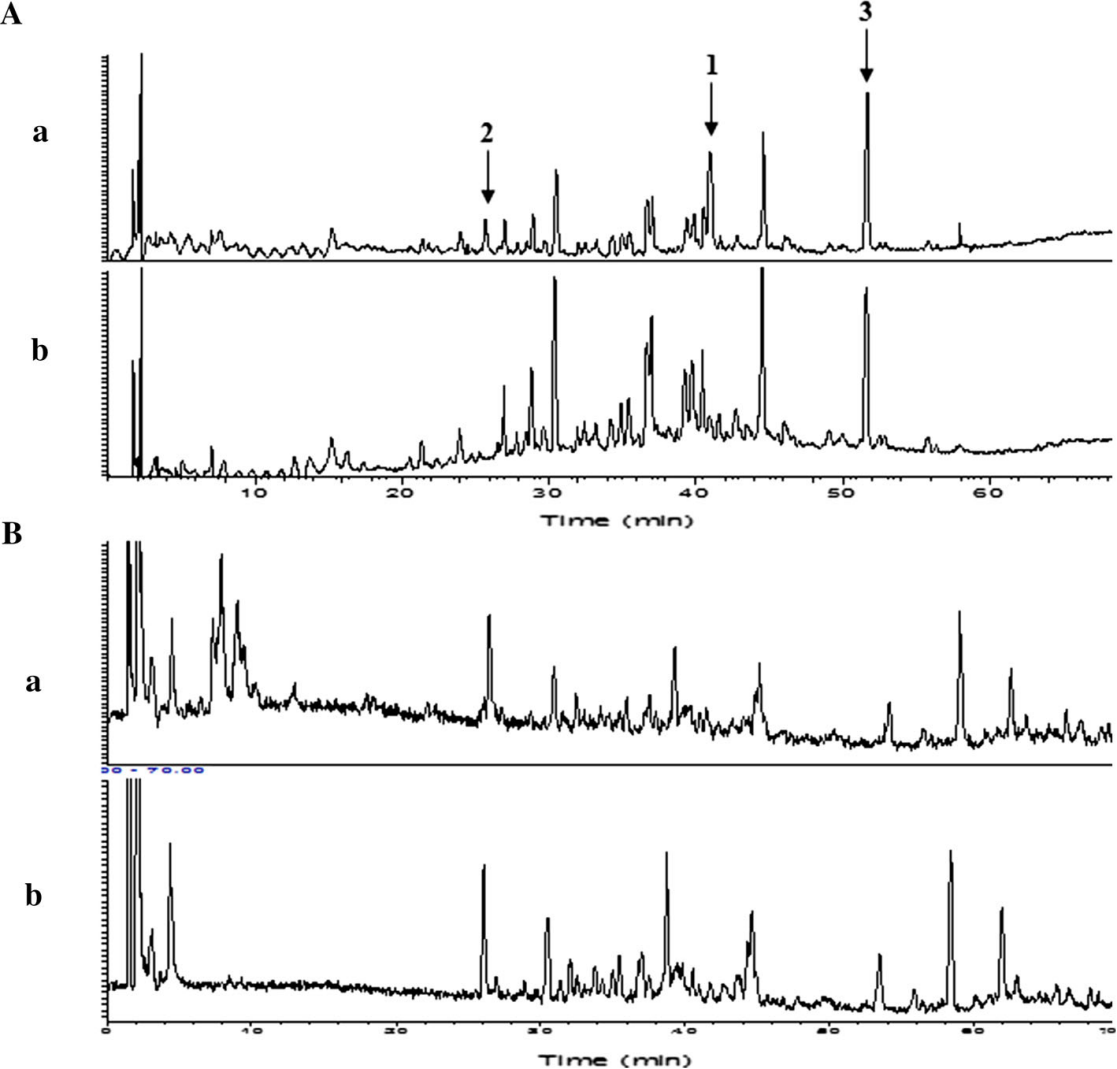
LC/Orbitrap MS analyses of *a* Ephedra Herb (EH) extract and *b* ephedrine alkaloids-free EH extract (*EFE*): **a** photodiode array (PDA, 254 nm) and **b** total ion chromatogram (TIC). Peak *1* 6-methoxykynurenic acid; peak *2* 6-hydroxykynurenic acid; peak *3* *trans*-cinnamic acid

**Fig. 3:**
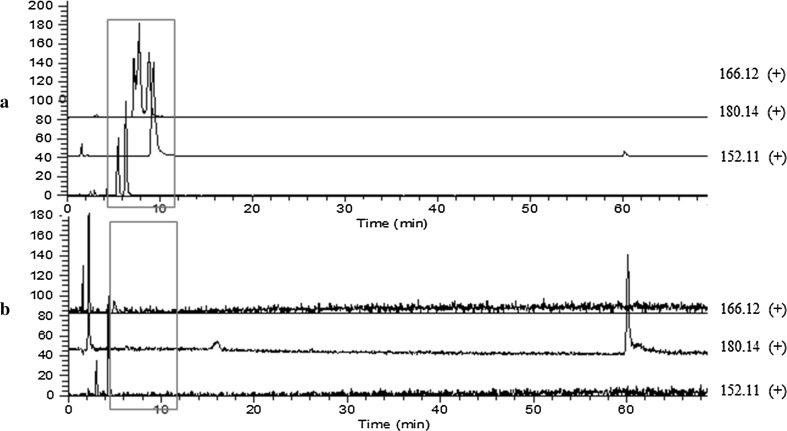
Extracted ion chromatograms of **a** Ephedra Herb (EH) extract and **b** ephedrine alkaloids-free EH extract (EFE) samples. *m*/*z* 166.12 [M+H]^+^, *dl*-ephedrine/*dl*-pseudoephedrine; *m*/*z* 180.14 [M+H]^+^, *dl*-methylephedrine/*dl*-pseudomethylephedrine; *m*/*z* 152.12 [M+H]^+^, *dl*-norephedrine/*dl*-pseudonorephedrine

**Fig. 4:**
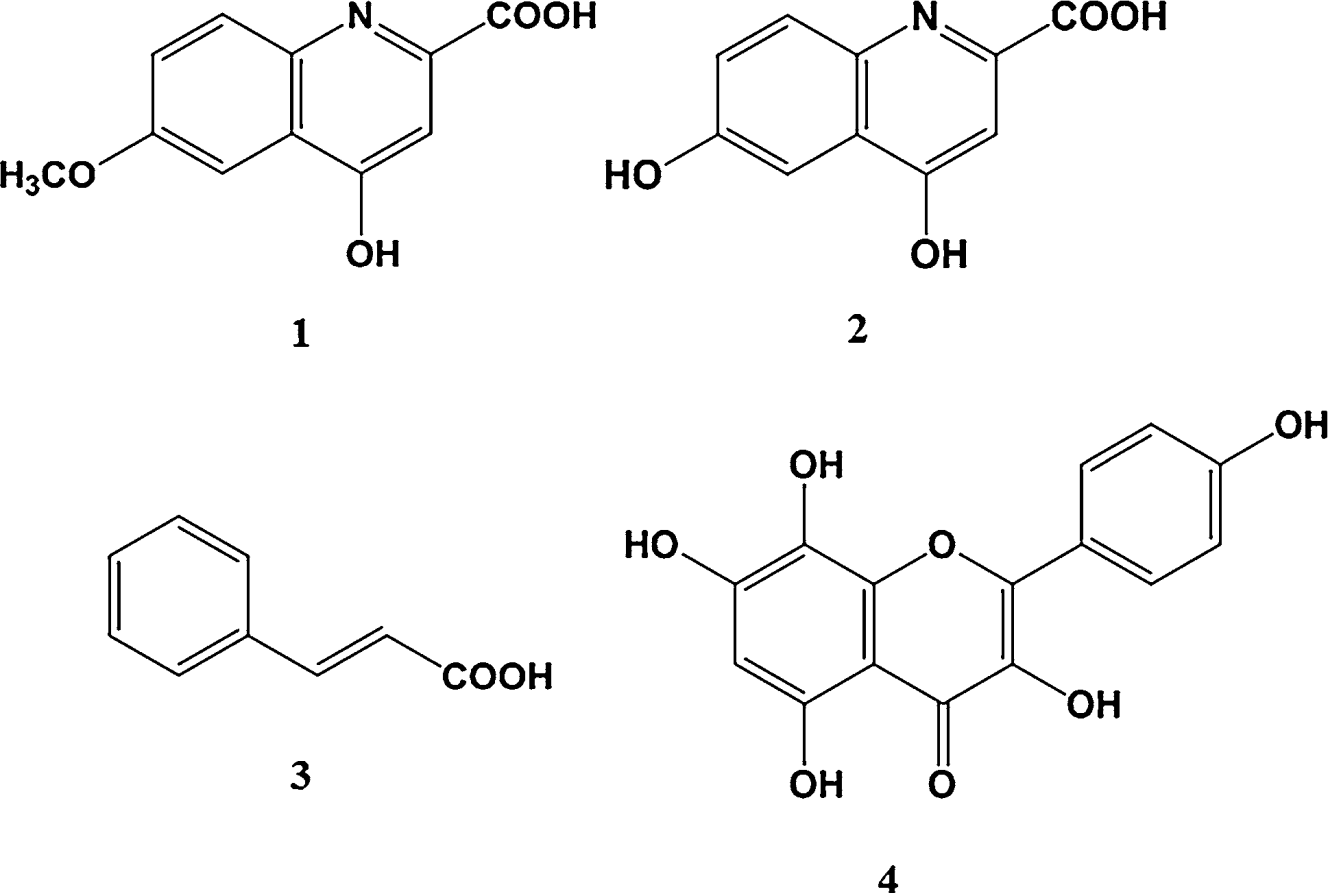
Structures of compounds **1**, 6-methoxykynurenic acid; **2**, 6-hydroxykynurenic acid; **3**, *trans*-cinnamic acid; **4**, herbacetin

To determine the chemical composition of the prepared EFE, we analyzed the EH extract and EFE using LC/Orbitrap MS (Fig. 2). The chromatograms of both were very similar, but the peaks around retention times 6–10 min and peaks 1 and 2 of the EH extract disappeared from that of EFE, while the height of peak 3 was slightly decreased. This observation was also supported by the LC/Orbitrap MS analysis of the adsorbed fraction on the cation exchange resin: the peaks mentioned above were detected in TIC or PDA (254 nm) chromatograms (Fig. 1S). The peaks that disappeared around retention times 6–10 min were identified as ephedrine/pseudoephedrine, methylephedrine/pseudomethylephedrine, and norephedrine/pseudonorephedrine by comparing their quasi-molecular ion peaks (*m*/*z* 166.12, 180.14, and 152.11) and retention times with those of authentic standard compounds (Fig. 3). Next, we examined the chromatograms to identify peaks 1–3. Previously, Starratt and Caveney [16] isolated 6-methoxykynurenic acid (**1**) and 6-hydroxykynurenic acid (**2**) from the cation exchange resin-treated fraction of *Ephedra pachyclada* subsp. *sinica*. The accurate determination of the masses of peaks 1 and 2 using Orbitrap MS analysis suggest that their molecular formulae were C_11_H_9_O_4_N and C_10_H_7_O_4_N, respectively. Therefore, we speculated that these peaks were those of **1** and **2**. Furthermore, the UPLC/MS analysis of the authentic sample of **1** showed a retention time and MS consistent with peak 1 (Fig. 2S). Therefore, we identified peak 1 as 6-methoxykynurenic acid (**1**, Fig. 4). Next, to identify peak 2, we synthesized **2** from the authentic sample of **1** using Kuo et al.’s method [17]. The product obtained was isolated and analyzed using NMR. The signal at *δ*_H_ 4.00 attributed to the 6-methoxy group in **1** had disappeared from the NMR spectrum of **2**, indicating that it was a demethylated form of **1**. The retention time and MS of **2** in the UPLC/MS analysis were the same as those of peak 2 (Fig. 3S). Therefore, we identified peak 2 as 6-hydroxykynurenic acid (**2**). Since peak 3 was hard to identify based on MS data, we separated the EH extract to identify it. Briefly, EH (100 g) was extracted with hot water (1 l) for 1.5 h. The extract (13.6 g) was fractionated by HP-20, CHP-20P, and silica gel column chromatography to get the fraction rich in peak 3. The NMR spectral signals of the fraction were observed at *δ*_H_ 7.75 (1H, d, *J* = 16.0 Hz), *δ*_H_ 6.45 (1H, d, *J* = 16.0 Hz), indicating a *trans*-double bond; *δ*_H_ 7.55 (2H, m), *δ*_H_ 7.40 (3H, m), indicating monosubstituted benzene; and *δ*_C_ 169.5, indicating carbonyl carbon of carboxylic acid, which speculated that the compound was *trans*-cinnamic acid (**3**). The UPLC/MS analysis of the authentic standard *trans*-cinnamic acid sample gave a peak with the same retention time and MS as those of peak 3 (Fig. 4S). Therefore, we identified peak 3 as *trans*-cinnamic acid (**3**, Fig. 4).

**Fig. 5:**
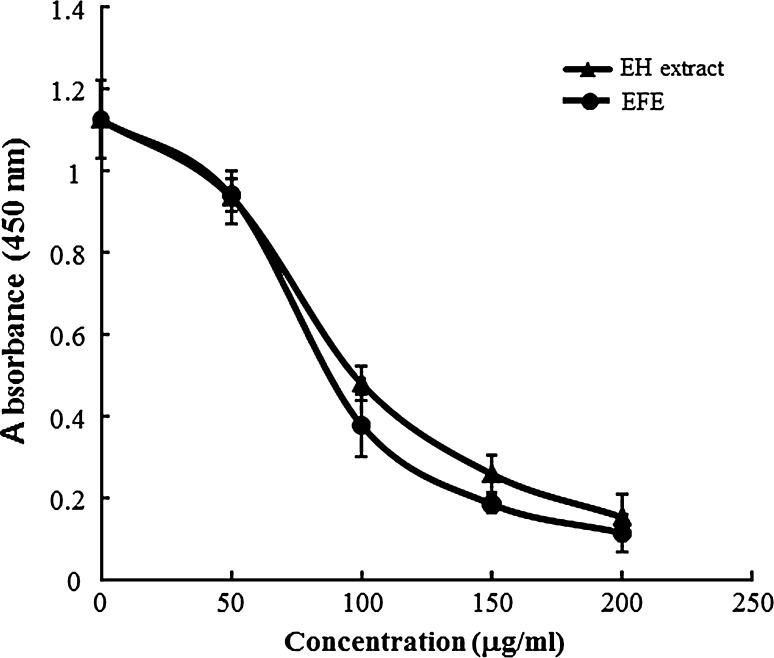
Ephedra Herb (*EH*) extract and ephedrine alkaloids-free EH extract (*EFE*) prevented proliferation of the H1975 non-small cell lung cancer (NSCLC) cell line

For the prepared EFE to satisfy quality standards for clinical use, a new stipulated quality control marker compound was required because EAs, the previously defined marker compounds for the quantitative assay of EH, are not present in EFE. Thus, we focused on herbacetin 7-*O*-neohesperidoside that was previously isolated from EH [15] and its aglycon, herbacetin, which have anticancer effects [13], and quantitatively determined their contents in EFE. First, we quantitatively analyzed herbacetin 7-*O*-neohesperidoside using the absolute calibration method. The quasi-molecular ion, *m*/*z* 609 [M–H]^−^, was detected using the SIM mode to obtain good linearity, accuracy, and precision in the concentration range of 1–50 μg/mL (Table 2). Subsequently, we analyzed herbacetin 7-*O*-neohesperidoside in EFE in sextuplicate using this condition (Fig. 5SA). The result revealed that the herbacetin 7-*O*-neohesperidoside content in EFE was 0.094 ± 0.009 %.

**Table 2:** Quantitative analysis of herbacetin 7-*O*-neohesperidoside and herbacetin

Compound	Monitoring ion (*m*/*z*)	Retention time (min)	Linear range (μg/ml)	Regression equation	*R* ^2^	Concentration (μg/ml)	Accuracy (%)	Precision (%)
Herbacetin 7-*O*-neohesperidoside	609	31	1–50	*y* = 15413*x* + 3746	0.9997	1	−4.28	15.7
						5	−22.6	16.2
						50	−7.22	9.87
Herbacetin	301	30	0.018–1.8	*y* = 0.4416*x* − 0.0122	0.9999	0.018	−1.20	1.22
						0.18	2.84	8.21
						1.8	8.43	2.64

Then, the total amount of herbacetin contained in the acid-hydrolyzed EFE was quantitatively determined using the internal reference method with apigenin (*m*/*z* 269 [M–H]^−^) as an internal standard. The quasi-molecular ion, *m*/*z* 301 [M–H]^−^, was detected using SIM to obtain good linearity, accuracy, and precision in the concentration range of 0.018–1.8 μg/ml (Fig. 5SB, Table 2). HCl (6 M) was added to the EFE sample, which was then stirred at 70 °C until the peaks corresponding to herbacetin 7-*O*-neohesperidoside and herbacetin 7-*O*-glucoside, which we previously isolated from EH, disappeared [14] in the LC/MS. Then, we analyzed the herbacetin content of the HCl-hydrolyzed EFE and found that it was 0.104 ± 0.002 %. These data showed that the sum of the herbacetin glycosides present in EFE was about 0.1 %, which appeared to be adequate for quantitative evaluation using HPLC. Herbacetin is a c-Met inhibitor found in EFE and is commercially available. These results suggest that it may be a suitable quality control marker compound for EFE.

Finally, to confirm whether the pharmacological effect was retained in the prepared EFE, we examined the effect of the EH extract and EFE on the growth of H1975 cells, and the result showed that they both prevented the proliferation of H1975 cells concentration-dependently (Fig. 5). The IC_50_ values of the EH extract and EFE were 88 and 76 μg/ml, respectively. These results revealed that the biological activity of EFE was retained and, therefore, the constituents that were removed during the manufacturing process did not affect its antiproliferative effect. Previously, we reported that EH impedes the HGF-induced motility of cancer cells by the inhibiting c-Met tyrosine kinase activity [10]. In addition, Tang et al. [18] reported that SU11274, a c-Met inhibitor, induced apoptosis of H1975 cells and inhibited their tumorigenesis in vivo. These reports suggest that EFE, as well as EH extract, suppressed the growth of H1975 cells through the inhibition of c-Met. Interestingly, EFE inhibited HGF-induced phosphorylation of c-Met and c-Met tyrosine kinase activity [19]. Therefore, EFE is expected to become a useful carcinostatic agent against cancer cells expressing c-Met. Furthermore, we have recently clarified that EFE shows analgesic and anti-influenza activity [19], suggesting that EFE has considerable therapeutic effects.

It has been reported that therapeutic regimens using Kampo medicines reduce the drug cost for patients compared with therapy using modern medicines [20–22]. Therefore, the relatively low cost of Kampo prescriptions is expected to have a considerable positive economic impact on cancer therapy, which frequently involves the use of expensive patented medicines. In the present study, EFE showed antiproliferative effects against cancer cells. Therefore, the clinical use of EFE derived from EH could not only expand the application range of EH, but could also contribute to reducing or, at least, limiting the use of expense anticancer therapies. Although EFE originated from EH, it is now a distinct medicine from EH. Therefore, drug approval for the clinical use of EFE as a new material will be required under the present drug regulation guidelines of Japan. However, no newly produced compounds were detected following the process used for EFE production from EH extract, which has been widely prescribed to Japanese patients for a long time. In addition, EAs, which are the main substance responsible for the side effects of EH, were effectively removed from EFE. Therefore, EFE is expected to be safer than EH. Future strategies for further developing EFE for clinical use involve the standardization of its quality. An efficient procedure for EFE production and quantitative determination of a marker compound, herbacetin, were demonstrated in this study, which could contribute to overcoming the limitations associated with the use of this herbal product.

## Conclusions

In this study, we established an efficient preparation method for Ephedrine alkaloids-free Ephedra Herb extract (EFE) from Ephedra Herb (EH) extract using a cation exchange resin, SK-1B. During the EFE production process, 6-methoxykynurenic acid and 6-hydroxykynurenic acid were also removed along with Ephedrine alkaloids (EAs), and the concentration of *trans*-cinnamic acid was slightly decreased. However, EFE showed antiproliferative effects similar to those of the EH extract, indicating that the removal of these constituents did not affect its biological activity. Furthermore, quantitative analyses of herbacetin in the EFE hydrolysate suggested that herbacetin could serve as a marker compound to control the quality of EFE for clinical use, although further studies are needed in order to clarify the pharmacological mechanisms underlying the activities of EFE. Moreover, the prepared EFE suppressed the growth of H1975 cells expressing c-Met. Therefore, EFE has the potential to become a useful carcinostatic agent against c-Met-expressing cancer cells without the adverse effects associated with EAs.

## Electronic supplementary material

Below are the links to the electronic supplementary material.

Supplementary material 1 (TIFF 78 kb) Fig. 1S. LC/Orbitrap MS analysis of the resin-adsorbed fraction: **a** photodiode array (PDA, 254 nm) and **b** total ion chromatogram (TIC). Peak *1*, 6-methoxykynurenic acid; peak *2*, 6-hydroxykynurenic acid; peak *3*, *trans*-cinnamic acid; *EAs*, ephedrine alkaloids.

Supplementary material 2 (TIFF 177 kb) Fig. 2S. Extracted ion chromatograms (XICs) of: **a** Ephedra Herb (EH) extract and **b** ephedrine alkaloids-free EH extract (EFE) and authentic standard (**1**) at *m*/*z* 220.

Supplementary material 3 (TIFF 190 kb) Fig. 3S. Extracted ion chromatograms (XICs) of: **a** Ephedra Herb (EH) extract and **b** ephedrine alkaloids-free EH extract (EFE) and synthetic compound (**2**) at *m*/*z* 206.

Supplementary material 4 (TIFF 161 kb) Fig. 4S. Extracted ion chromatograms (XICs) of: **a** Ephedra Herb (EH) extract and **b** ephedrine alkaloids-free EH extract (EFE) and authentic standard (**3**) at *m*/*z* 149.

Supplementary material 5 (TIFF 134 kb) Fig. 5S. Analyses of: **A** herbacetin 7-*O*-neohesperidoside and **B** herbacetin. *a* Extracted ion chromatogram (XIC) at *m*/*z* 609 of ephedrine alkaloids-free Ephedra herb extract (EFE), *b* XIC at *m*/*z* 609 of herbacetin 7-*O*-neohesperidoside, *c* XIC at *m*/*z* 301 of EFE, *d* XIC at *m*/*z* 301 of herbacetin.

## Abbreviations

EH: Ephedra Herb
EFE: Ephedrine alkaloids-free Ephedra Herb extract
EA: Ephedrine alkaloid
