# Supplementary figures and images for: Efficiently prepared ephedrine alkaloids-free Ephedra Herb extract: a putative marker and antiproliferative effects
Source: J Nat Med. 2016 Mar 14;70:554–62. doi: 10.1007/s11418-016-0977-1 (PMC4935757; doi:10.1007/s11418-016-0977-1)

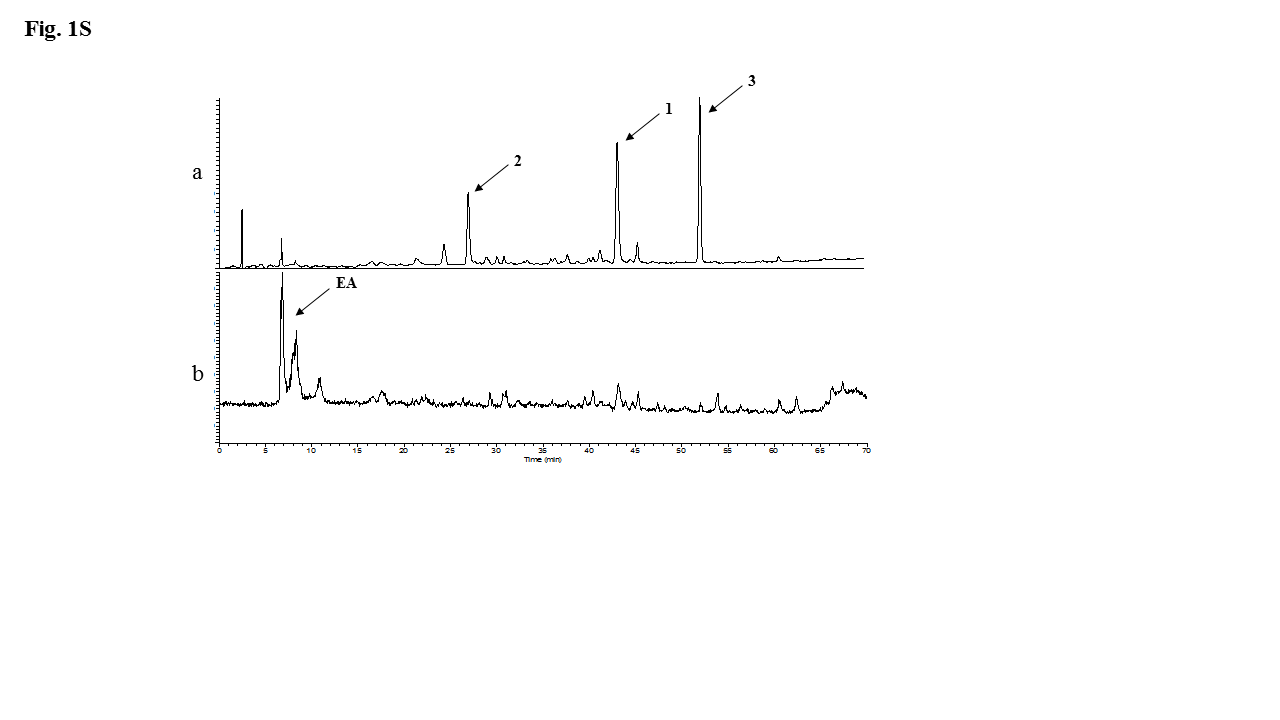

Supplement: Supplementary file 1 — Supplementary material 1 (TIFF 78 kb) Fig. 1S. LC/Orbitrap MS analysis of the resin-adsorbed fraction: a photodiode array (PDA, 254 nm) and b total ion chromatogram (TIC). Peak 1, 6-methoxykynurenic acid; peak 2, 6-hydroxykynurenic acid; peak 3, trans-cinnamic acid; EAs, ephedrine alkaloids. [file 11418_2016_977_MOESM1_ESM.tiff]

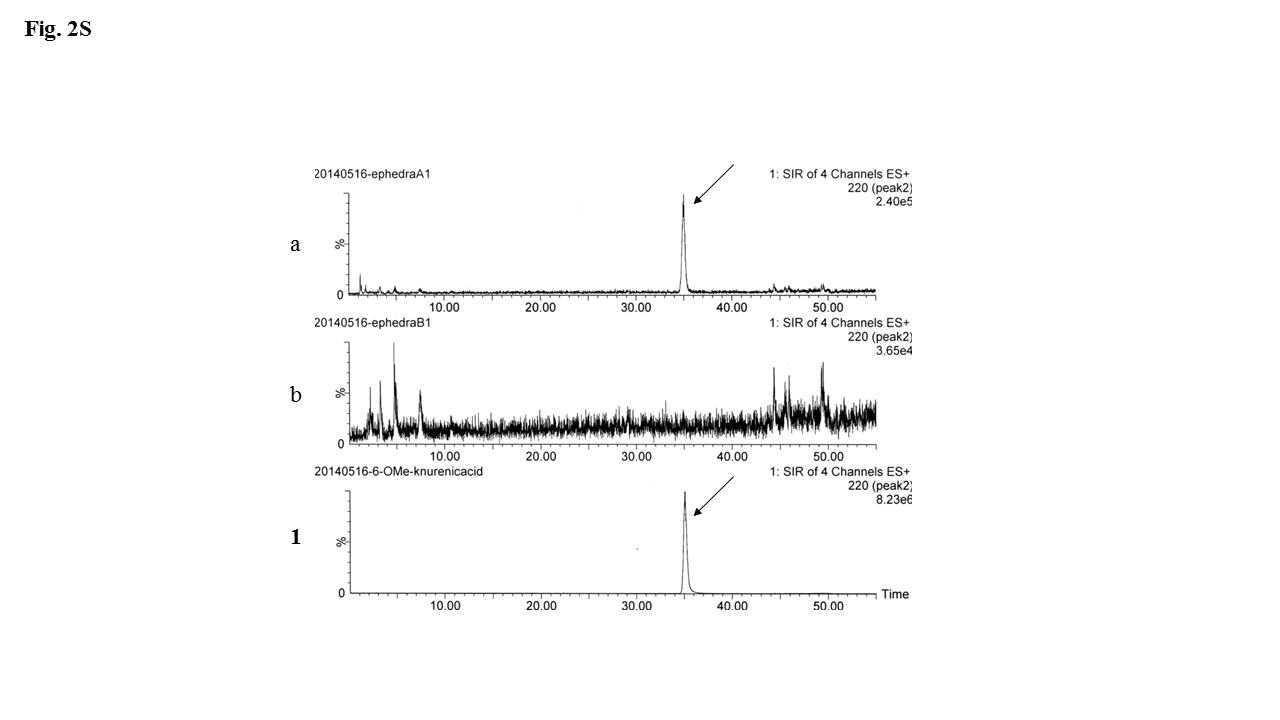

Supplement: Supplementary file 2 — Supplementary material 2 (TIFF 177 kb) Fig. 2S. Extracted ion chromatograms (XICs) of: a Ephedra Herb (EH) extract and b ephedrine alkaloids-free EH extract (EFE) and authentic standard (1) at m/z 220. [file 11418_2016_977_MOESM2_ESM.tiff]

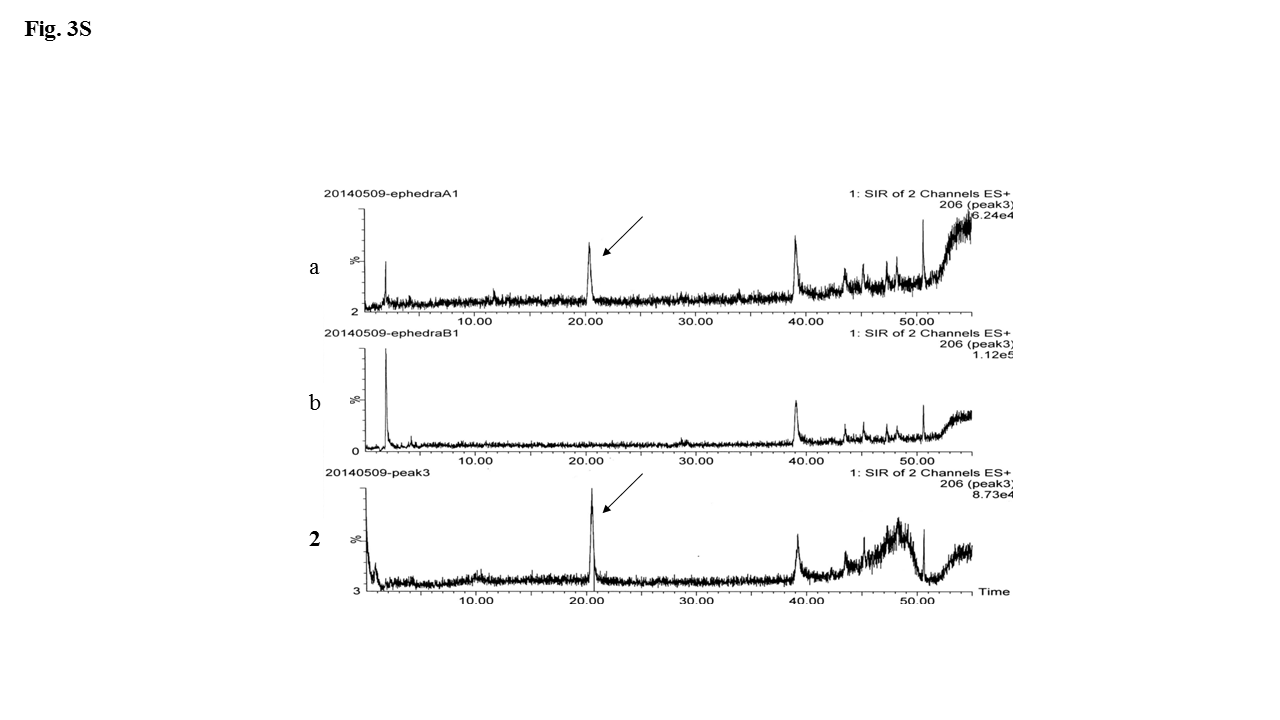

Supplement: Supplementary file 3 — Supplementary material 3 (TIFF 190 kb) Fig. 3S. Extracted ion chromatograms (XICs) of: a Ephedra Herb (EH) extract and b ephedrine alkaloids-free EH extract (EFE) and synthetic compound (2) at m/z 206. [file 11418_2016_977_MOESM3_ESM.tiff]

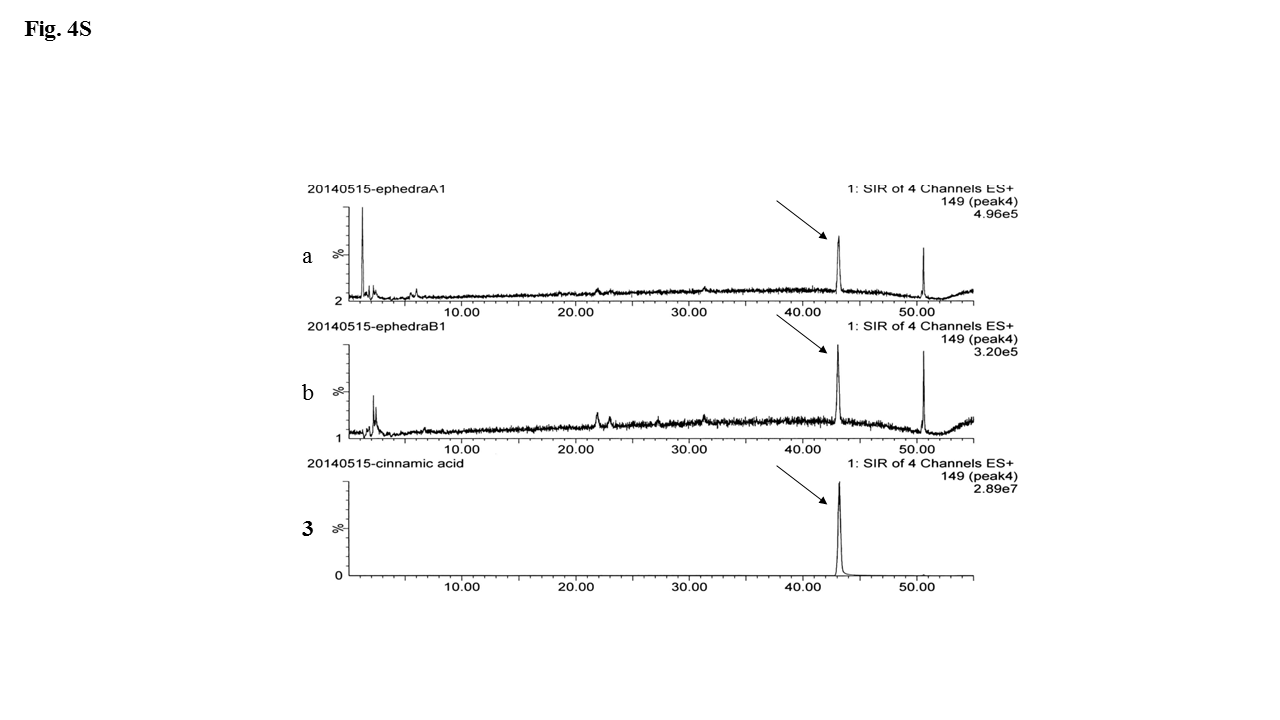

Supplement: Supplementary file 4 — Supplementary material 4 (TIFF 161 kb) Fig. 4S. Extracted ion chromatograms (XICs) of: a Ephedra Herb (EH) extract and b ephedrine alkaloids-free EH extract (EFE) and authentic standard (3) at m/z 149. [file 11418_2016_977_MOESM4_ESM.tiff]

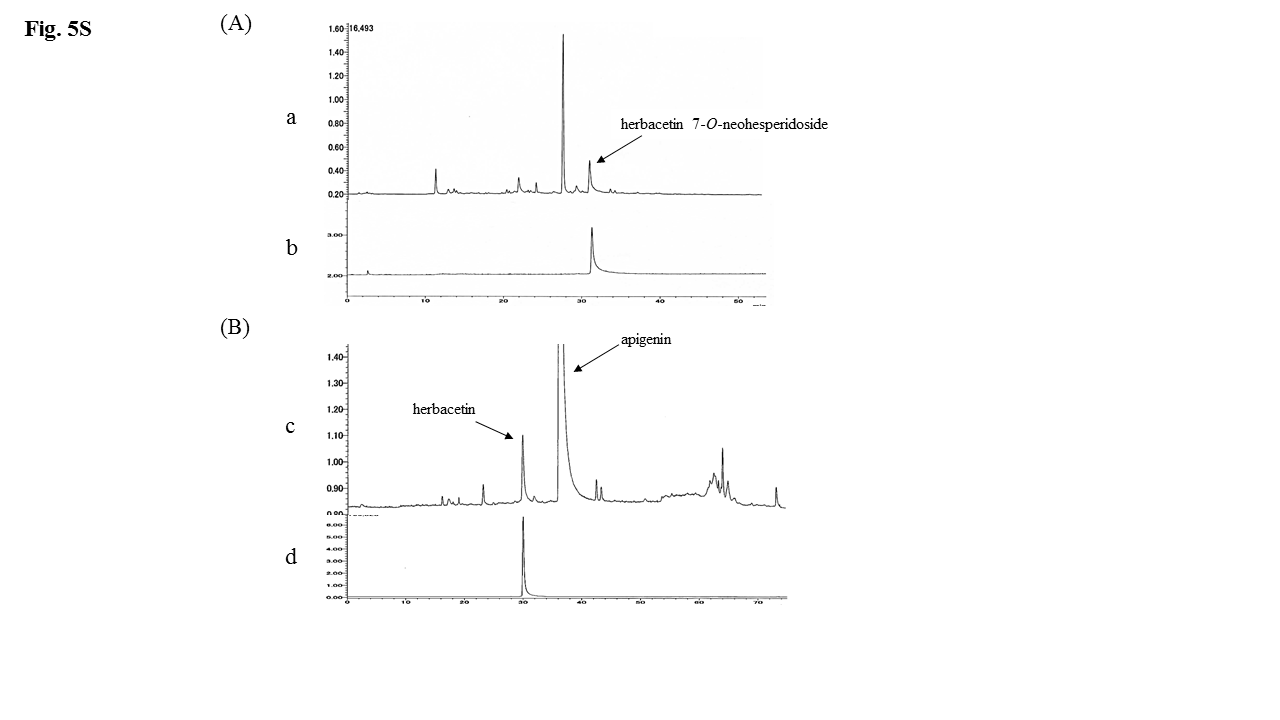

Supplement: Supplementary file 5 — Supplementary material 5 (TIFF 134 kb) Fig. 5S. Analyses of: A herbacetin 7-O-neohesperidoside and B herbacetin. a Extracted ion chromatogram (XIC) at m/z 609 of ephedrine alkaloids-free Ephedra herb extract (EFE), b XIC at m/z 609 of herbacetin 7-O-neohesperidoside, c XIC at m/z 301 of EFE, d XIC at m/z 301 of herbacetin. [file 11418_2016_977_MOESM5_ESM.tiff]
